# Cloning and Characterisation of *Schistosoma japonicum* Insulin Receptors

**DOI:** 10.1371/journal.pone.0009868

**Published:** 2010-03-24

**Authors:** Hong You, Wenbao Zhang, Malcolm K. Jones, Geoffrey N. Gobert, Jason Mulvenna, Glynn Rees, Mark Spanevello, David Blair, Mary Duke, Klaus Brehm, Donald P. McManus

**Affiliations:** 1 Queensland Institute of Medical Research, Brisbane, Queensland, Australia; 2 School of Population Health, University of Queensland, Brisbane, Queensland, Australia; 3 School of Veterinary Science, University of Queensland, Brisbane, Queensland, Australia; 4 School of Tropical Biology, James Cook University, Townsville, Queensland, Australia; 5 Institute für Hygiene und Mikrobiologie, Universität Würzburg, Würzburg, Germany; New England Biolabs, United States of America

## Abstract

**Background:**

Schistosomes depend for growth and development on host hormonal signals, which may include the insulin signalling pathway. We cloned and assessed the function of two insulin receptors from *Schistosoma japonicum* in order to shed light on their role in schistosome biology.

**Methodology/Principal Findings:**

We isolated, from *S. japonicum,* insulin receptors 1 (SjIR-1) and 2 (SjIR-2) sharing close sequence identity to their *S. mansoni* homologues (SmIR-1 and SmIR-2). SjIR-1 is located on the tegument basal membrane and the internal epithelium of adult worms, whereas SjIR-2 is located in the parenchyma of males and the vitelline tissue of females. Phylogenetic analysis showed that SjIR-2 and SmIR-2 are close to *Echinococcus multilocularis* insulin receptor (EmIR), suggesting that SjIR-2, SmIR-2 and EmIR share similar roles in growth and development in the three taxa. Structure homology modelling recovered the conserved structure between the SjIRs and *Homo sapiens* IR (HIR) implying a common predicted binding mechanism in the ligand domain and the same downstream signal transduction processing in the tyrosine kinase domain as in HIR. Two-hybrid analysis was used to confirm that the ligand domains of SjIR-1 and SjIR-2 contain the insulin binding site. Incubation of adult worms *in vitro*, both with a specific insulin receptor inhibitor and anti-SjIRs antibodies, resulted in a significant decrease in worm glucose levels, suggesting again the same function for SjIRs in regulating glucose uptake as described for mammalian cells.

**Conclusions:**

Adult worms of *S. japonicum* possess insulin receptors that can specifically bind to insulin, indicating that the parasite can utilize host insulin for development and growth by sharing the same pathway as mammalian cells in regulating glucose uptake. A complete understanding of the role of SjIRs in the biology of *S. japonicum* may result in their use as new targets for drug and vaccine development against schistosomiasis.

## Introduction

Schistosomes are parasitic blood flukes infecting approximately 200 million people globally [1] of which 20 million have severe disease; 250,000 deaths are directly or indirectly attributable each year to schistosomiasis [2]. Praziquantel is highly effective in curing individuals with schistosomiasis but the high rates of re-infection after treatment limit its role in control [3]. Consequently, inclusion of a vaccine in combination with chemotherapy and/or other suitable interventions will be required for sustainable control of schistosomiasis [4]. The tegument, the outermost surface of the intra-mammalian stages of schistosomes, is a dynamic host-interactive layer involved in nutrition, immune evasion, excretion and signal transduction. Apical membrane proteins expressed on the surface of the schistosomulum and adult worm are logical targets on which to focus for both vaccine and drug development [5].

As sensing and responding to environmental factors are essential in the complex life cycle of schistosomes, studying on signal transduction molecules and their functional mechanisms will be necessary for elucidating schistosome host-parasite interactions and parasite biology [6], [7], [8]. Notably, the highly adapted relationship between schistosomes and their hosts appears to involve exploitation by the parasite of host endocrine and immune signals [9], [10]. Receptor tyrosine kinases (RTKs) are high affinity cell surface receptors that bind many ligands including hormones, growth factors, cytokines, and trigger different signalling cellular cascades for the control and regulation of cell proliferation and differentiation [11]. One important RTK is the insulin receptor that is activated by insulin [12].

Diverse molecular pathways dependent on kinase signalling have been described in schistosomes and shown to be involved in the host-parasite relationship [8]. *Schistosoma mansoni* epidermal growth factor receptor (SER), which contains a conserved intracellular tyrosine kinase domain and an extracellular domain for binding epidermal growth factor (EGF) ligands, was the first RTK described in schistosomes [13]. It has been demonstrated that human EGF can bind SER and induce SER phosphorylation and activation of a conserved Ras/ERK-dependent signalling pathway [8], [13]. Moreover, human EGF was shown to increase protein and DNA synthesis as well as protein phosphorylation of primary sporocysts of *S. mansoni in vitro*, indicating a possible role for EGF signaling in the host-parasite relationship and in parasite development [14]. Recently, the characterisation of two types of transforming growth factor-beta (TGF-β) type I (SmTβRI) and II (SmTβRII) receptor of *S. mansoni* [15], [16], demonstrated that in the presence of human TGF-β1, SmTβRII was able to activate SmTβR1, which in turn activated the TGF-β signaling pathway. This pathway could play an important role in the development of vitelline cells in the female worm, via a stimulus from the male schistosome, and in embryogenesis, given that the components of TGF-β signaling localise in the tegument, which is the interface between male and female schistosomes [17].

Transcriptomic and proteomic profiling has revealed that, similar to their mammalian counterparts, schistosomes encode a panel of growth factors and cytokines including EGF-like peptides and fibroblast growth factor (FGF)-like peptides, as well as receptors for thyroid and steroid hormones [18], [19], [20], implying that schistosomes can accept host hormone signals for cell proliferation, development, mating, and reproduction, in addition to responding to endogenous parasite endocrine hormones by sharing high identity with mammalian orthologues [21], [22].

Insulin is a key hormone which has profound effects on both carbohydrate and lipid metabolism, and has a significant influence on protein (increases amino acid transport into cells) and mineral metabolism. We showed recently, using microarray analysis, that 1,101 genes of adult *S. japonicum* were up- or down-regulated in response to insulin; these genes were involved predominantly in males worms in the promotion of protein synthesis and the control of protein degradation, as observed in classical mammalian systems, and in female fecundity through up-regulation of genes associated with the mitogenic-activated protein kinase (MAPK) sub-pathway [23]. It has been demonstrated that *S. japonicum* shares sequences with its mammalian host for insulin receptor or insulin-like growth factor receptor and can accept host hormone signals for development [18], [24].

The first parasitic helminth insulin receptor (IR) was isolated from *Echinococcus miltilocularis* [25], and two distinct IRs (SmIR-1, SmIR-2) were identified subsequently in *S. mansoni* [26]. SmIR-1 and SmIR-2 belong to the family of IRs containing a conserved catalytic domain, and sequence analysis showed they share a conserved α2β2 heterotetramer structure with those of *E. multilocularis* IR (EmIR) and HIR [26]. The parasite IRs were able to bind human insulin [25], [26], supporting earlier reports showing that parasites can use host insulin or insulin-like growth factors to regulate and control growth and development [27], [28]. Based on the sequence of the EmIR gene, one type of IR was also amplified by reverse transcriptase-polymerase chain reaction (RT-PCR) from both *Taenia crassiceps* and *Taenia solium* [29]; these IRs have been implicated in the establishment, growth and reproduction of these cestodes by exploitation of the host endocrine system [30], [31].

In the present work, we describe the presence of two discrete IRs in *S. japonicum* (SjIRs), an analysis of their relationship with other IRs, assays showing the binding ability of the SjIRs with human insulin, and the incubation of adult worms *in vitro* both with a specific insulin receptor inhibitor and anti-SjIRs antibodies. Understanding the roles of SjIRs in *S. japonicum* will reveal new information on the molecular interchange between host and parasite that may provide a keystone for the future design of a drug or vaccine that would exclusively bind to the parasite IR, blocking the insulin effect and the signal transduction pathways mediated by the hormone.

## Materials and Methods

### Ethics statement

All work was conducted with the approval of the Queensland Institute of Medical Research Animal Ethics Committee.

### Parasites

*S. japonicum* adult worms were collected by perfusion of ARC Swiss mice or rabbits infected percutaneously with 40 or 1000 cercariae of *S. japonicum* (Anhui population, mainland China), respectively, shed from *Oncomelania hupensis* snails as described [32].

### Cloning *S. japonicum* insulin receptors

A Qiagen RNeasy kit (Qiagen, Hilden, Germany) was used to purify total RNA from adult *S. japonicum*. A one step RT-PCR (Qiagen) kit was employed to amplify specific cDNA. Based on the conserved regions of the catalytic domains of IRs in *S. japonicum* and *S. mansoni* and partial *S. japonicum* sequences available at http://function.chgc.sh.cn/sj-proteome/index.htm, four pairs of primers for *SjIR-1* and five primer pairs for *SjIR-2* (Table S1) were designed to obtain full-length cDNA sequences by an overlap strategy of PCR amplification. The cDNA sequences obtained were then used in BLAST analysis of the *S. japonicum* transcriptome and proteome database (http://function.chgc.sh.cn/sj-proteome/index.htm) to search for 5′ and 3′ terminal sequences.

### Sequence and phylogenetic analyses

Sequence alignment was performed using Biomanager (http://www.biomanager.angis.org.au) and Clustal W program (http://align.genome.jp/), with default parameters. The alignment of intracellular regions was manually modified in order to take into account the presence of inserts in the tyrosine kinase (TK) domains of SjIRs, SmIRs and EmIR. Structural analysis was performed using BLAST (http://www.ncbi.nlm.nih.gov/BLAST/Blast.cgi?), SMART (http://smart.embl-heidelberg.de.), ScanProsite (http://www.expasy.org/tools/scanprosite/) and MotifScan (http://myhits.isb-sib.ch/cgi-bin/motif_scan), and the Clustal W program for sequence alignments.

For phylogenetic analyses, alignments were generated that included additional IR sequences (restricted to the 11 kinase sub-domains of the TK domain or to the L1, cysteine-rich and L2 sub-domains of the ligand domain). Phylogenetic trees were produced in MrBayes [33]. The Jones model for amino-acid substitution [34] + G (gamma distribution of rates with four rate categories) + I (proportion of invariant sites) was used. Values for I and for the shape parameter (alpha) for the gamma distribution were estimated from the data during the runs. Two million generations (2 runs each of 4 chains) were specified for each alignment and the chain was sampled every 1000 generations. The first 500 trees were discarded as “burnin”. Only regions where alignment was regarded as reliable were used in the input file for MrBayes. For the TK domain, 235 sites were used. For the ligand domain, 315 sites were used.

### Structure prediction using homology modelling and the crystal structure of the human IR

The three dimensional-structures of the insulin receptor domains of SjIR-1 and SjIR-2 were predicted based on the crystal structure of the insulin receptor domain of the murine insulin receptor (PDB code 2DTG) using the program Modeller [35]. The structures of the TK domains of SjIR-1 and SjIR-2 were predicted in the same fashion using the TK domain crystal structure of the HIR (PDB code 1IRK). In both cases, 50 models were calculated and the model with the lowest objective function score selected as the representative model. Alignments for the model, generated using the Modeller align function, were altered manually to provide the best representative alignment. The stereochemical quality of the modelled structure was analysed using procheck [36]. Structure comparisons and figure generation were achieved using Pymol or VMD (visual molecular dynamics) [37].

### Yeast two hybrid analyses

Yeast two hybrid analyses was employed to determine whether the SjIRs were able to bind human insulin *in vivo* and used the Gal4-based MATCHMAKER™ system (Clontech, Mountain View, USA) according to the manufacturer's instructions. For constructing translational fusions to the Gal4-activation domain (AD), we inserted the ligand domain sequence of SjIR-1 (LD1, encoding protein sequence from D59 to E411) or SjIR-2 (LD2, from Q7 to K399) into the vector pGADT7 after the fragments were amplified by PCR using adult *S. japonicum* cDNA as template with primers SjIR-1-TF1(5′-GGCATATGGACTGTTCCGGACGTTTACTGAATTTAC-3′) and SjIR-1-TR1 (5′-CTGCAGCTTCACAATCACGAATACTAATAAGGATTGATTG-3′); or SjIR-2-TF2 (5′-GGCATATGCAACATGATTCAACAGATCTCGGTTCC-3′) and SjIR-2-TR2 (5′-CTGCAGTTTTCAAATTTTTAAACGCTCTGTCCAATAAGTTAG-3′) via *Nde*I and *Pst* I restriction sites incorporated into the primer sequences. A positive control was constructed with the ligand binding domain of the HIR (from Cys-8 to Ser-340) and a negative control was constructed with the C-terminal region of the *E. multilocularis* ERM (ezrin/radixin/moesin)-like protein homologue (ElpC) [25], [26]. These controls were used as indicators of reaction for the binding assay. In order to construct translational fusions to the Gal4 DNA binding domain (BD), a DNA fragment encoding human pro-insulin was inserted into the GBKT7 vector via *Nde*I and *Eco*RI restriction sites as described [25], [26].

All vector constructs were confirmed as having correct reading frames by DNA sequencing. The AD vector [containing SjIRs ligands (LD1 and LD2), HIR ligand and negative fragment, respectively] and BD vector containing human pre-insulin fragment were transformed into yeast strain AH109. Interaction trap analysis of diploids resulting from the mating of AH109 was performed according to the MATCHMAKER™ instructions using low stringency (SD/-Leu/-Trp), medium stringency (SD/-His/-Leu/-Trp) and high stringency (SD/-Ade/-His/-Leu/-Trp/X-α-gal) agar plates. Formation of colonies was assessed after 3 days of incubation at 30°C. Positive colonies were picked for genomic DNA extraction and purification using a Y-DER Yeast DNA extraction Reagent Kit (Pierce, Rockford, IL, USA). Hot-PCR (Qiaqen) was used to amplify the LD1 or LD2 and human pre-insulin fragments from the DNA of the same colony with the specific primers for the pGADT7(T7 and 3′AD primers) and GBKT7 (T7 and 3′ BD primers) vectors. Bands of the correct size were analysed by DNA sequencing.

### Production of antibodies

Antibodies were raised against the ligand domains of the SjIRs and were produced in mice against the N-terminal fragments of SjIR-1 (from D59 to E411) (termed LD1) and SjIR-2 (from R37 to C525) (termed LD2). Forward (5′-GCATATGGACTGTTCCGGACGTTTACTGAATTTACGT-3′) and reverse (5′-CGGAGCTCGTTCACAATCACGAATACTAATAAGGATTG-3′) primers for LD1 and forward (5′-GGGATCCGCGTTGCACTGTCATAGAAGG-3′) and reverse (5′-GCTCGAGTCACCAATTACAATAAGCTAAATCTCCATTTGT-3′) primers for LD2 were used for amplification and cloning into the pET28c and pET28b vectors (Novagen, Madison, USA), respectively, and transformed into *Escherichia coli* (BL21 strain) for expression induced with 1mM IPTG (isopropyl thio-β-D-galactoside) at 37°C for 3h. Recombinant proteins were purified by chromatography using a Ni-NTA His-tag affinity kit (Novagen) under denaturing conditions using 6M guanidine according to the manufacturer's instructions. To produce anti-sera, mice were immunized subcutaneously with 20 µg recombinant protein in complete Freund's adjuvant as primary immunisation, and given 3 boost immunisations at intervals of two weeks with the same quantity of protein emulsified with incomplete Freund's adjuvant. Bloods for sera were collected two weeks after the last boost.

To generate antibodies against the TK domains of the SjIRs, we used a truncated strategy to express soluble peptides having a relatively high antigenicity based on analysis by MacVector 8.0. We combined the C-terminal peptide fragments of SjIR-1 (from P1248 to S1268, PASQQILIDTNGNGRESTANS; from D1295 to Y1300, DIREFY; and from A1341 to A1363, ATYLRQQMSKDDCPHGSIEPKLA) termed as T1; and the C-terminal fragments of SjIR-2 (from I1590 to P1609, IDEDPSCDSNGRNFDPTNDP; from 1204 to E1209, RDFINE; and from T1620 to A1659, TTSSSKSSPIFNADRDYKTHQITCNSSEDTSVNLNDNRVA) termed as T2. The synthesised T1 and T2 cDNA fragments were inserted into the pET41b vector (Novagen) and transformed into *Escherichia coli* (BL21 strain) for expression as GST fusion proteins induced with IPTG. Recombinant proteins were purified by chromatography using a Ni-NTA His-tag affinity kit (Novagen) according to the manufacturer's instructions. The process of generating anti-T1 and T2 antibodies in mice was identical to that described above for LD1 and LD2.

All the anti-T1, T2 and LD1, LD2 antisera were adsorbed against lysates of bacterial cells transformed with appropriate pET expression vector expressing the GST or His tag to remove anti-GST or - His antibodies [38].

### Western blot analysis

Anti-T1, T2 and LD1, LD2 antisera were used to detect the four purified recombinant proteins and to probe native T1, T2 and LD1, LD2 proteins in crude *S. japonicum* extracts as follows: 1) 30 ng of recombinant T1, T2 and LD1 or LD2 protein was mixed in the same volume of loading buffer, separated on SDS/PAGE gels, and then transferred onto nitrocellulose membrane. 2) Crude adult worm antigen was prepared from adult worms of *S. japonicum* freshly perfused from rabbits percutaneously infected with 1000 cercariae six weeks previously. After five washes in PBS to minimise contamination of the schistosome protein extracts with host components, the worms were homogenised on ice in 20 mM Tris-buffer (PH 7.4) containing 1 mM EDTA and a protease inhibitor cocktail (Sigma, Castle Hill, Australia), and the homogenate was then centrifuged at 16,000 g for 1 h at 4°C. The supernatant was collected as soluble adult worm antigen preparation (SWAP) and stored at −70°C. The pellet was suspended and, after 2 washes with PBS, was dissolved in 1% (w/v) SDS in the same volume as that of the SWAP. The preparation was then centrifuged at 16,000 g for 15 min at 25°C, the supernatant collected as insoluble protein, and stored at −70°C. For Western blot analysis, the soluble and insoluble protein preparations were mixed separately in the same volume of loading buffer, separated on SDS/PAGE gels and then transferred onto nitrocellulose membrane.

After blocking in 5% (v/v) milk in PBS, the membrane was incubated overnight at 4°C with mouse anti-T1 or -T2 (dilution 1∶50) or anti-LD1 or -LD2 (dilution 1∶100) anti-sera, respectively. After 3 washes with PBS containing 0.05% (v/v) Tween-20, the membrane was soaked in goat anti-mouse HRP-conjugate IgG (Bio-Rad) (1 in 2000 dilution) and incubated at 37°C for 1 h. Following washing (5 x), detection of immunoreactive bands was obtained using 4-chloro-1-naphanol substrate solution.

### Inhibitor treatment and *in vitro* receptor blocking assays using anti-LD1, LD2 anti-sera

Adult worms of *S. japonicum* were freshly perfused with warm RPMI (37°C) (Invitrogen, Carlsbad, USA) from mice, 7 weeks post-infection with 40 cercariae. Following washing (3x) in the same medium, worms were incubated in RPMI1640 supplemented with 100 U/ml penicillin, 100 ug/ml streptomycin and with 20% (v/v) foetal bovine serum (as a source of insulin) (Sigma) at 37°C in an atmosphere of 5% CO_2_ in air overnight. The worms were then cultured in two ways:

(1). In the presence or absence (control) of 100 µM insulin receptor inhibitor- HNMPA-(AM)3 (Hydroxy-2-naphthalenylmethylphosphonic acid trisacetoxymethylester) (BIOMOL, Hamburg, Germany) for 30 min, after which glucose levels in worms of different groups were determined. Worm aliquots were homogenised in 20 mM Tris/Hcl buffer for 2–4 min on ice, then boiled for 5 min, centrifuged at 15,800 *g* for 30 min at 4°C, and the supernatants used to measure the concentration of protein by the Bio-Rad protein assay (Bio-Rad, Mississauga, Canada). The glucose levels of worms (ng glucose/µg protein) were measured using a glucose assay kit (Sigma) according to the manufacturers' protocols. Samples of worms were also used for extraction of total RNA in order to determine the transcription levels of the *GTP4* gene by real time PCR analysis (details below).

(2) In the presence of: A) With 10% (v/v) anti-LD1 +10% anti-LD2 anti-sera; or B) With 20% (v/v) normal mouse serum (control). To inactivate complement, each serum was incubated at 56°C for 30 min before use. Cultures were established at 37°C in 24-well culture plates with each well having 3 ml RPMI 1640 containing anti-serum and 10 worms. The culture medium was gassed with a mixture of 5% CO_2_ in air. The worms in each group were collected after 24 h culture with the different sera. Glucose levels and transcription levels of the *GTP4* gene of worms were determined for each group as described above.

The statistical significance of differences between HNMPA-treated and control groups in glucose level and *GTP4* transcription level were calculated using t-test of repeated measures. The statistical significance of differences between anti-sera treated and control groups (0 h and 24 h) in glucose level and *GTP4* transcription level were calculated using one-way ANOVA of repeated measures. P values≤0.05 were considered statistically significant. GraphPad Prism software was used for all statistical analyses.

### Quantitative RT-PCR

*SjIR-1* and *SjIR-2* transcript levels were determined in different developmental stages of *S*. *japonicum* using real-time (RT)-PCR. Total RNA was isolated separately from miracidia, sporocysts, cercariae, schistosomulum, and male and female adult worms. First strand cDNA was synthesized from total RNA using a Sensiscript Reverse Transcription for First-stand cDNA synthesis Kit (QIAGEN) with oligo(dT)15 primers. Forward and reverse primers (Sigma-Aldrich) were designed for *SjIR-1*: SjIR-1F 5′-GCCTCATTACCATATCCTGGTT-3′ and SjIR-1R 5′- GCTGGTTCAAATTCCCAACA-3′; and *SjIR-2*: SjIR-2F 5′-TGGTGTTGTTTTGTGGGAGA-3′ and SjIR-2R 5′-GCCATTGGAGAGTAGCGTTT -3′. NADH-ubiquinone reductase was employed as a housekeeping gene [39]. The cDNA samples were diluted to 50 ng/µl, quantified by a ND-1000 spectrophotometer (Nano Drop, Wilmington, USA), and then divided into 5 µl of aliquots at 1∶10 dilution. The reaction was combined with 10 µl SYBR Green (Applied Biosystems, Warrington, UK), 3 µl of water and 2 µl (2.5pmol) of the forward and reverse primers in a 0.1 ml tube and loaded on a Rotor-Gene-6000 (Corbett Research, Sydney, Australia) for PCR amplification. Data were analysed using Rotor Gene 6 software (Corbett Research). Parameters were set by determination of the primer melting temperature and addition of a melt curve to show primer viability. Each experiment was performed in duplicate and the confidence threshold (CT) of the second set was normalised to the first set before evaluation. For graphical representation of qPCR data, copy numbers, obtained for the different stages, were deducted from the CT values.

*SjIR-1* and *SjIR-2* transcripts were also quantified in different female worm tissues (gastrodermis, ovary and vitelline glands) using real time PCR with the same primers as above. A PALM microbeam laser catapult microscope was used to microdissect the gastrodermis, ovary and vitelline tissue from frozen female worm sections [40]. Sections were scraped from the slide using a sterile scalpel blade for RNA extraction using RNA aqueous-Micro kits (Ambion, Austin, USA). First strand cDNAs were synthesized from the total RNA for real time PCR analysis as described above.

*GTP4* transcripts were quantified in cultured worms using the forward and reverse primers (Sigma-Aldrich): GTP4F 5′-TAAGCTCTTTACTCAGAAAGATTTACGTATGC-3′ and GTP4R 5′- AACAACACAAAACTGTATGTAGTCAAGTGGGAT-3′.

### Immunolocalisation

Horseradish peroxidise (HRP) labelling was used for the immunolocalisation of SjIR-1 and SjIR-2. Freshly perfused adult male and female worms were fixed in 100% methanol, washed again in PBS, then embedded in Tissue-Tek Optimal Cutting Temperature (OCT) compound (ProSciTech, Queensland, Australia) and sectioned with a cryostat into 7.0-µm sections. Frozen sections were fixed in ice cold acetone for 5 min followed by drying and then rehydrating in PBS for 5 minutes. Endogenous peroxidise activity was blocked by incubating the sections in 3% H_2_O_2_ 0.1% sodium azide in PBS for 10 min followed by 3 washes with PBS for 5 min each. Non-specific antibody binding was inhibited by incubating the section in 10% (v/v) normal goat serum in PBS for 30 min; then the sections were incubated with mouse anti-T1 (1∶25 diluted in PBS) or anti-T2 anti-serum (1∶50 diluted in PBS) for 1 h at room temperature. After 3 washes with PBS, the slides were incubated with anti-mouse HRP (DAKO, Glostrup, Denmark) at room temperature for 30 min. After a further 3 washes with PBS, colour was developed for 5 min with a NovaRED subtract Kit (VECTOR laboratories, Burlingame, USA), which produces a positive red reaction product.. Sections were slightly counterstained in Mayer's haematoxylin, then dehydrated, cleared, mounted and scanned using an Aperio scanner (Aperio, Vista, USA).

## Results

### *S. japonicum* has two different insulin receptors with features of *S. mansoni* insulin receptors

Based on the highly conserved sequences of *IR*s for *S. mansoni* [26] and *E. multilocularis* [25], we designed primers (listed in Table S1) and used PCR to amplify the fragments by an overlap strategy (see details in Materials and Methods) using *S. japonicum* adult worm cDNA as template. Two types of *IR* cDNA were isolated from *S. japonicum*, *SjIR-1* and *SjIR-2*. *SjIR-1* has a complete cDNA sequence and comprises an open reading frame (ORF) of 4,599 bp (GenBank accession number GQ214553) encoding 1,533 amino acids (aa). We used a number of different strategies to amplify the distant 3′ terminal sequence of *SjIR-2* and were able to obtain the complete sequence except the last exon of 21bp shown to be presented in the *S. japonicum* transcriptome and proteome database (http://function.chgc.sh.cn/sj-proteome/index.htm). *SjIR-2* contains an ORF of 5,096 bp (GenBank accession number GQ214554) encoding 1,698 aa. Protein sequence comparisons confirmed SjIR-1 and SjIR-2 encode insulin receptors.

SjIR-1 and SjIR-2 have 70% and 74% identity to SmIR-1 and SmIR-2, respectively. The two receptor sequences have similar features/domains to the insulin receptors from *S. mansoni*, *E. multilocularis*, *Homo sapiens* and *Drosophila*, in both the extracellular ligand domain (LD) (Figure S1A) and intracellular tyrosine kinase (TK) domain (Figure S1B). Domains or sub-domains of SjIR-1 and 2 and sequence identities with other IRs of *S. mansoni*, *E. multilocularis* and *Homo sapiens* are are shown in Table S2.

**Figure 1:**
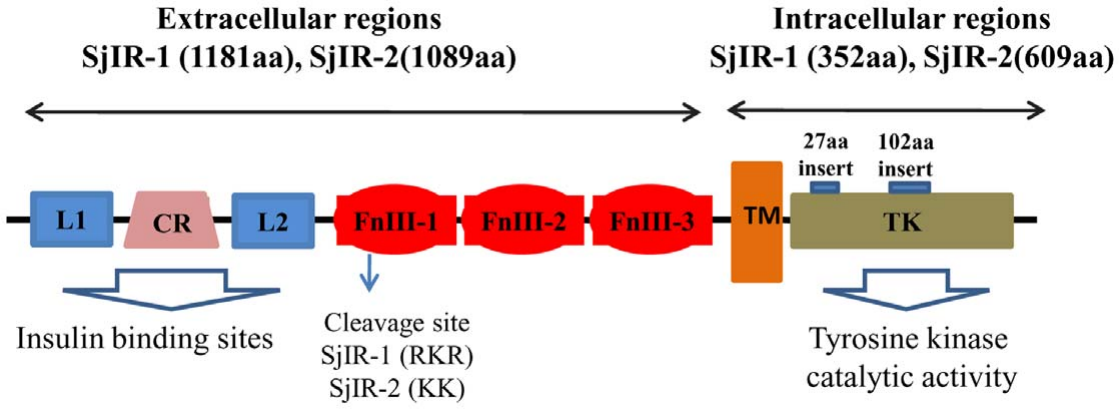
Domain structure of the SjIRs. Analysis of the amino acid sequences for the SjIRs predicted all the characteristic domains of IRs from other taxa, including ligand binding loop 1 (L1) and loop 2 (L2); cysteine-rich region (CR), fibronectin type III domain (FnIII-1, FnIII-2, FnIII-3); transmembrane domain (TM); and a tyrosine kinase domain (TK). The position of the putative cleavage site is marked with an arrow. The coding regions for the 27aa insert in SjIR-1 and the 102aa insert in SjIR-2 are marked with a blue bar.

Similar to IRs from *S. mansoni*, *E. multilocularis*, *Homo sapiens* and *Drosophila* (Figure S1A, S1B), SjIR-1 or SjIR-2 is composed of two α-subunits and two β-subunits (α_2_β_2_ structure). The α-subunits are extracellular and contain the insulin ligand domain, which is responsible for binding. The N-terminal of the extracellular domain contains two loops (L1 and L2) separated by a cysteine-rich region (CR) shown in Figure 1. The C terminus of the extracellular domain of the SjIRs is formed from three Fibronectin 3 type (FnIII) repeats; FnIII-1, FnIII-2 and FnIII-3 (shown in Figure 1) [39], [40]. The β subunits of the SjIRs extend through the transmembrane (TM) domains and contain a TK domain, which is responsible for kinase activity. The intracellular region of SjIR-1 and SjIR-2 comprises a TK domain containing 11 typical sub-domains (from I to XI, Figure S1B) as in other taxa (Figure S1B). The TK domains of SjIR-1 and SjIR-2 have 40.8% and 44.6% identity with the HIR TK domain, respectively (Table S2). The domains of the SjIRs have all the highly conserved key motifs described in SmIRs for tyrosine kinase activity [41] and these are presented in detail in Figure S1B. SjIR-2 has the similar “three tyrosine residues” (YxxxYY1413, Figure S1B) involved in the recruitment of SH2 [42] to HIR, EmIR, DmIR and SmIR-2, whereas SjIR-1 (or SmIR-1) has lost the first Y in this region. The NPXY1132 binding motif of the IR (Figure S1B), was also found in SjIR-2 or SmIR-2, but not in SjIR-1 or SmIR-1.

Alignment analysis showed a short insert of 27 amino acids (SQQILIDTNGNGRESTANSKHVISLGS1276) is present in the TK domain of SjIR-1 (with 52.4% identity to SmIR-1, which also contains an insert of 29 aa) between sub-domain I and II when compared with HIR. The insert contains no tyrosine residues or known motifs. MacVector™ 8.0 analysis (data not shown) predicted this insert region to be highly antigenic and hydrophilic. Further comparison of SjIR-2 and HIR indicated the presence of an insert of 102 amino acids in the SjIR-2 TK domain (with 56.9% identity to SmIR-2, which also contains an insert of 110 aa) between sub-domains IV and V; this is equivalent to the 172 amino acid stretch described by Konrad et al [25] in the EmIR sequence (Figure S1B), which has no similarity with the 102 amino acid insert in SjIR-2. This insert is highly antigenic and has a helical structure (data not shown; analysis by MacVector™ 8.0). Similar to the SmIRs, the C-terminal tail of SjIR-2, without known or predicted functions, is much longer than that of SjIR-1.

Overall, the sequence analysis showed clearly that the predicted functions of IR-1 and IR-2 in *S. japonicum* and *S. mansoni* are likely to be similar.

### SjIR2 has a more complex gene structure than SjIR1

We compared the cDNA sequences of *SjIR-1* and *SjIR-2* with genomic sequences (http://function.chgc.sh.cn/sj-proteome/index.htm) and recognised their exon-intron genomic structures (Figure S1C). *SjIR-1* and *SjIR-2* are composed of 23 and 25 exons, respectively. Exon sizes in *SjIR-1* vary between 54 bp and 588 bp (with an average of 200±122 bp) whereas exon sizes in *SjIR-2* range between 20bp and 617 bp (with an average of 204±120 bp). Similar complexity is evident for *SmIR-1* (24 exons), *SmIR-2* (25 exons), *HIR* (22 exons), and *IGF-1* receptor (21 exons) genes [26]. Two exon/introns in *SjIR-1* and one exon/intron in *SjIR-2* are missing compared with genome sequence data presented for *S. japonicum* at http://function.chgc.sh.cn/sj-proteome/index.htm. The sizes of introns were variable with the average sizes being 2414±1616bp and 1464±1067bp for *SjIR-1* and *SjIR-2*, respectively. The G+C% intronic content is 33.5% ±2% and 33.2%±3% for *SjIR-1* and *SjIR-2*, respectively, similar to that in most other sequenced genomes of the Metazoa [43]. There are no repeating or overlapping sequences in either genomic sequences, suggesting *SjIR-1* and *2* are single copy genes in *S. japonicum*.

### Homology modelling recovered a conserved structure between the SjIRs and HIR

**Figure 2:**
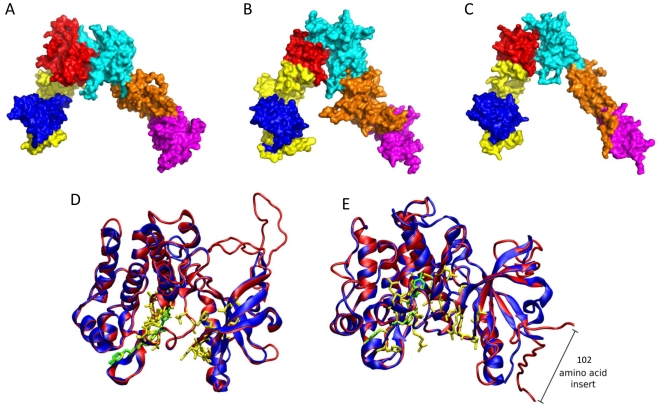
Molecular models of the ligand domain and tyrosin kinase domain of SjIR-1 and SjIR-2. Surface rendering of LD of SjIRs and HIR. A, Surface rendering of LD of SjIR-1; B, Surface rendering of LD of SjIR-2; C, Surface rendering of LD of HIR. Individual sub-domains are coloured as follows: L1, blue; CR, yellow; L2, red; FnIII-1, cyan; FnIII-2, orange; FnIII-3, magenta Molecular models of the TK domains of SjIR-1 (D) and SjIR-2 (E) (red ribbons) are superimposed on the template structure of the TK domain of HIR (blue ribbon) (PDB code 1IRK). Conserved motifs are highlighted in stick representation; specific residues (ATP binding site, sequence required for ATP stabilisation, motif implicated in phosphotransfer and Mg^2+^ binding site) responsible for tyrosine kinase activity are coloured in yellow and the autophosphorylation sites in green. Superimposition was produced using the program VMD and the STAMP structural alignment tool [37].

Amino acid sequences of SjIR-1 and SjIR-2 have 12.0–40.8% and 17.3–44.6% identity to HIR in the different sub-domains (Table S2), respectively. To identify whether the low identity of SjIR significantly impacts on the functional structure, we used a homology modelling program to calculate the changes in the protein structure of SjIR. Homology models for the insulin receptor domain of SjIRs were calculated using the crystal structure of the IR-domain of the murine insulin receptor. The models of the ligand domains of SjIR-1, SjIR-2 and HIR are shown in Figure 2A, 2B, 2C, respectively. Although model quality was affected by insertions and differences in the parasite molecules (See Figure S1A), each α-β monomer of SjIR-1 (Figure 2A) and SjIR-2 (Figure 2B) has an inverted “V” layout which is similar to HIR (Figure 2C) with respect to the cell membrane. As shown in Figure 2(A-C), one leg of the V is formed by the L1 (marked in blue), CR (yellow) and L2 (red) domains; the other is formed by an extended linear arrangement of the three FnIII domains (FnIII-1, marked in cyan; FnIII-2, orange; FnIII-3, magenta).

The overlay of the TK domain of HIR and SjIR-1 is shown in Figure 2D and the overlay of HIR and SjIR-2 is shown in Figure 2E. The model quality for these domains was superior to that for the IR domains and the models adopted a similar fold to characterised TK domains, including the positioning of conserved residues important for function, such as Tyr1189 and 1190 in HIR, which are the most striking features of the 1IRK structure and are autophosphorylated upon insulin binding the active site of the enzyme. The SjIR-2 TK domain has a substantial insert (102 aa) between domains IV and V. The amino acid sequence of this insert was not sufficiently similar to any other protein in the Protein Data Bank so that its structure could not be predicted using homology modelling methods; the conserved regions of the SjIR-2 TK domain adopted a structure similar to the SjIR-1 TK domain (Figure 2D and 2E).

### Phylogenetic analyses of SjIR-1 and SjIR-2 showing their evolutionary relationships

Figure S2 (A and B) shows the phylogenetic relationships of SjIRs and SmIRs with various invertebrate and vertebrates based on the protein sequences of the TK domain and LD, respectively. As expected from the levels of sequence identity, both LD and TK domains for SjIR-2, SmIR-2 and EmIR were grouped on a branch different from that of SjIR-1 and SmIR-1. The phylogenetic positions of SjIRs are similar to those of the SmIRs [26]. Despite the evolutionary divergence of vertebrates and invertebrates, the essential features of the structure and intrinsic functions of the insulin receptor are remarkably conserved [44]. Comparative sequence analysis of the SjIRs, SmIRs and EmIR, with HIR showed that the TK domain is more conserved than the LD domain. In the latter domain, SjIR-1 has 74%, 23.6% and 18.9% identity with SmIR-1, EmIR and HIR, respectively, whereas SjIR-2 has 75.7%, 37.1% and 17.1% identity with SmIR-2, EmIR and HIR, respectively. In the TK domain, SjIR-1 has 83.3%, 43% and 37.7% identity with SmIR-1, EmIR and HIR, respectively, with SjIR-2 having 83.8%, 49.5% and 29.3% identity with SmIR-2, EmIR and HIR, respectively.

### Significant binding ability of SjIR-1 and SjIR-2 with human insulin *in vivo*

To test the binding ability of SjIR-1 and SjIR-2 to human insulin, we employed yeast two hybrid analysis to identify their binding interaction *in vivo*. The ligand binding domains (LD) of SjIR-1 and SjIR-2 were fused to the Gal4 activation domain (Gal4AD), respectively and tested for interaction with human pro-insulin fused to the Gal4 DNA binding domain (Gal4BD). The LD of HIR and the C-terminal region of the *E. multilocularis* ERM (ezrin/radixin/moesin)-like protein homologue (ElpC) [25], [26] were used, respectively, as positive and negative LD controls. The Figure S3 shows that the different receptor LDs were able to interact *in vivo* (in yeast) with human pro-insulin at medium stringency; none of the receptor LDs, including HIR were able to bind insulin at high stringency conditions. SjIRs showed similar insulin binding ability to HIR and SmIRs [26]. To identify whether the binding was because the hybridisation system contained target sequences (see details in the Materials and Methods) in positive colonies, we used PCR to amplify both the human insulin and *SjIR* sequences in one colony. Figure S3 shows that both genes were presented in the system. We confirmed right sequences of human insulin and SjIRs by sequencing (data not shown).

### Comparative expression of SjIR-1 and SjIR-2

**Figure 3:**
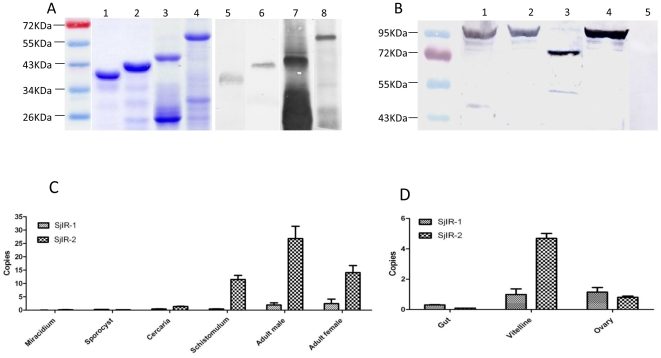
Expression of SjIR-1 and SjIR-2. (A) SDS-PAGE gel stained with Coomassie Brilliant Blue showing purified recombinant proteins T1 (lane 1), T2 (lane 2), LD1 (lane 3) and , LD2 (lane 4). Western blot analysis using anti-T1 (lane 5), T2 (lane 6), LD1 (lane 7) and LD2 (lane 8) antibodies to detect the appropriate purified fusion protein. (B) Western blot analysis of total extracts from adult *S. japonicum* worms (lanes 1, 2, 3, 4, 5). Protein extracts were detected by antibodies raised in mice against T1 (lane 1), T2 (lane 2), LD1 (lane 3), LD2 (lane 4) and pre-immune sera (lane 5). (C) Quantification of transcripts in different developmental stages of *S. japonicum* - miracidium, sporocyst, cercaria, schistosomulum, adult male and adult female; (D) Quantification of transcripts in the gut, vitelline tissue and ovary of female *S. japonicum*. Quantitative amplification of SjIR-1 and SjIR-2 cDNA was performed in triplicate. NADH (Contig7836) was used as a housekeeping gene [39].

To generate specific antibodies against SjIR-1 and SjIR-2, we used cDNAs encoding two combined polypeptides (termed T1 for SjIR-1 and T2 for SjIR-2) from the TK domains and the N-terminal fragments of SjIR-1 (termed LD1) and SjIR-2 (termed LD2) to insert into a pET vector, respectively for expression (see details in Materials and Methods). Fragments of T1, T2, LD1 and LD2 were expressed as fusions in *E. coli* and the recombinant proteins were purified (Figure 3A). The intact protein and an amount of degraded product of approximately half the size of SjLD1 were visualised. T1 contained a partial sequence of the additional 27 aa insert between sub-domains I and II of the TK domain discussed earlier to increase the specificity of SjIR-1. The T2 fragment was expressed with truncated sequences with high antigenicity and hydrophobicity from the extra C-terminal tail of SjIR-2 as described above. The specificity of each antiserum was tested by probing each purified recombinant antigen (Figure 3A). Western blot analysis showed that affinity purified T1, T2 and LD2 contained a single band with the predicted size of the recombinant fusion-protein; LD1 showed a single band of about 45KDa with a smear of bands below 30KDa, most likely degradation products (Figure 3A). These results provided confirmation of the ability of the anti-sera to recognise the corresponding recombinant proteins.

Western blot analysis showed the anti-T1, T2, LD1and LD2 anti-sera also recognised components in a crude extract of adult *S. japonicum* worms (Figure 3B). Both the anti-T1 and anti-T2 antisera recognised bands of approximately 96KD which matches well with the calculated molecular weights for T1 (96 KD) and T2 (98KD), based on their protein sequences and estimated cleavage sites, which separate the α and β chains [45]; the anti-LD1 and anti-LD2 antisera recognised bands of approximately 76KD and 92KD, respectively. Bands recognised by anti-SjIR-1 and SjIR-2 antisera correspond to the putative cleavage sites RKR857 in SjIR-1 and KK839 in SjIR-2. Control sera from pre-immunized mice did not reveal recognition of any *S. japonicum* protein component.

Real-time PCR was used to quantity the transcription levels of *SjIR-1* and *SjIR-2* in different life cycle stages of *S. japonicum*. Both receptors were transcribed in the mammalian host stages with high expression in male and female adult worms compared with schistosomulum (Figure 3C). A similar level of transcription of *SjIR-1* was evident in males and females but very low *SjIR-1* transcription occurred in schistosomulum and cercariae. *SjIR-2* expression was elevated in males with transcription levels in female worms and schistosomulum being similar; a low level of *SjIR-2* transcription was detectable in cercariae. Overall, *SjIR-2* was more abundantly expressed in *S. japonicum* than *SjIR-1*. Neither *SjIR-1* nor *SjIR-2* was expressed in measurable levels in sporocysts or miracidia.

Real time PCR was also used to quantity the transcription levels of *SjR-1* and *SjR-2* in three different tissues (gastrodermis, ovary and vitelline glands) of adult female *S. japonicum*. *SjIR-1* had a higher transcription level in the vitelline tissue and ovary than in the gut (Figure 3D). *SjIR-2* was also more highly expressed (5X) in the vitelline grands compared with the ovary; no transcription of *SjIR-2* was detected in the gut. Overall, the transcription level of *SjIR-2* was higher than that of *SjIR-1* in females.

### Inhibiting and blocking the SjIRs result in significantly decreased worm glucose consumption

**Figure 4:**
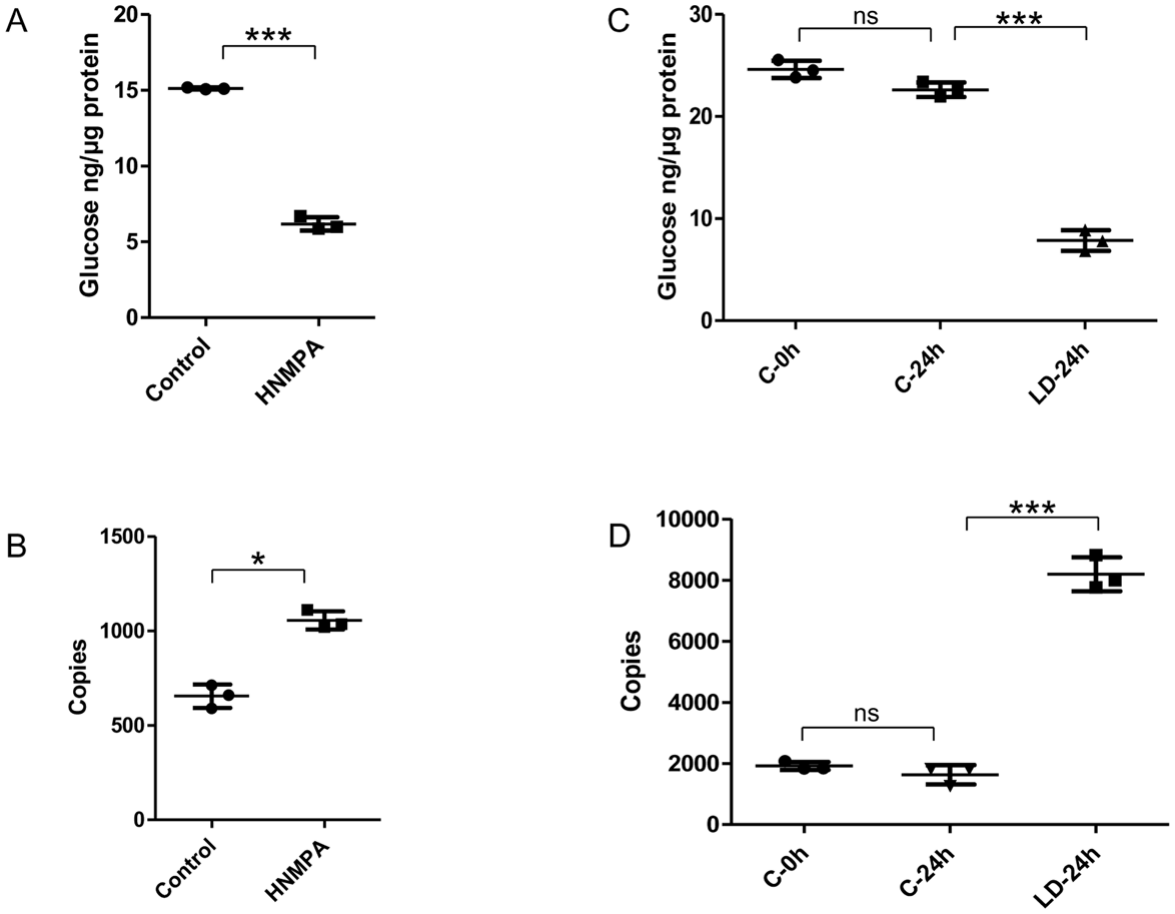
Inhibitor treatment and *in vitro* receptor blocking test using anti-SjIRs antibodies. A: Glucose levels of worms cultured with HNMPA inhibitor (100 µM) for 30 min; B: *GTP4* transcription levels of worms cultured with HNMPA for 30 min; C: Glucose levels of worms cultured with 20% anti-LD1+LD2 anti-sera for 24 h; D: *GTP4* transcription levels of worms cultured with 20% anti-LD1+LD2 anti-sera for 24 h. Data are means ±SD (shown as error bars) of triplicates from a typical experiment. A t-test was used to make the comparison between the HNMPA-treated and control groups, and one-way ANOVA was used to make the comparison between the anti-sera treated and control groups. P value≤0.05*, P value≤0.001**, P value≤0.0001***.

We used an insulin receptor TK inhibitor (HNMPA) and antibodies against the LDs of the SjIRs to inhibit/block their function and to determine their role in glucose consumption by *S. japonicum*. Real time PCR was employed to determine whether the inhibition/blocking could impact on the transcription of glucose transporter 4 (*GTP4*), the indirect target protein of IR. Incubation with HNMPA (100 µM) for 30 min resulted in a reduction in glucose levels of 40% in worms (P value = 0.0006), compared to the control groups (Figure 4A). After 30 min cultivation with HNMPA, the transcription level of GTP4 was increased 1.6 times in worms (P value = 0.022) compared with the control group (Figure 4B).

Culture of *S. japonicum* for 24 h with 20% anti-LD1+ anti-LD2 anti-sera resulted in a 65% reduction in glucose (P≤0.0001) compared with the level in worms cultured in medium containing normal serum (Figure 4C). There was no significant change in glucose level in the control group (incubated with normal serum over the 24 h incubation period). Real time PCR showed that the transcription level of GTP4 in worms cultured with anti-LD1+LD2 anti-sera for 24 h was increased 5 times (P≤0.0001) compared to that of worms prior to the addition of the anti-sera (Figure 4D). There was no significant change in the transcription level of GTP4 in the control group incubated with normal serum over the 24 h incubation period.

### SjIR-1 and SjIR-2 have different locations in *S. japonicum* adult worms

**Figure 5:**
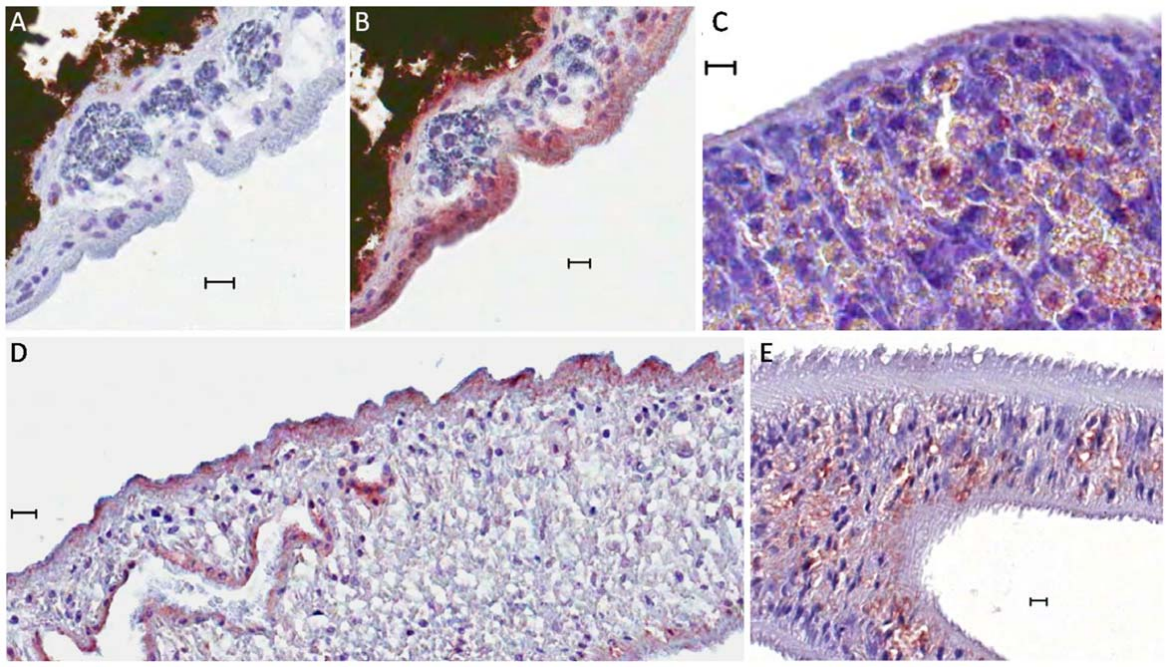
Horseradish peroxidise labelling showing the immunolocalisation of SjIR-1and SjIR-2 in adult male and female worms. Worm sections were labelled with mouse anti-T1 (B and D) or anti-T2 (C and E) anti-serum coupled with DAKO anti-mouse HRP and scanned using an Aperio scanner. Negative control sections were incubated with mouse pre-immune sera (A). Scale bars are in 10 µm. SjIR-1 was detected on the tegument basal membrane, in muscle and in the intestinal epithelium of adult female (B) and male (D) worms. SjIR-2 was localized in the vitelline cells of females (C) and the parenchyma of males (E).

Figure 5 shows the localisation of SjIR-1 and SjIR-2 in adult male and female worms, probed using the anti-T1 and anti-T2 antisera described above. SjIR-1 was predominantly localized in the tegument basal membrane, muscles and the intestinal epithelium of female (Figure 5B) and male worms (Figure 5D), which was similar to that observed for SmIR-1 [26]. SjIR-2 was localized in the vitelline tissue of female worms (Figure 5C) and the parenchyma of males (Figure 5E). In contrast, SmIR-2 was shown to be localized in the parenchyma of both female and male worms [26]. No labelling was evident with control sera from pre-immunized mice (Figure 5A).

## Discussion

Metazoans ranging from cnidarians to chordates employ insulin-like hormones and corresponding receptors for intercellular communication [25]. Insulin receptors have also been isolated from *E. multilocularis* (EmIR) [25] and *S. mansoni* (SmIR-1 and SmIR-2) [26]. We successfully isolated two IR cDNAs from *S. japonicum*. We used sequence analysis and binding affinity studies to confirm that the sequences encoded insulin receptors. SjIR-2 and SmIR-2 are phylogenetically closer to EmIR than to SjIR-1 and SmIR-1, indicating that SjIR-1 and SmIR-1 might perform specific functions in schistosomes, while SjIR-2 (with higher similarity to HIR), SmIR-2 and EmIR might share similar roles in parasite growth and development in the three parasitic flatworms. A BLAST search in GenBank detected only a partial sequence with similarity to SjIR-2 and SmIR-2 in *Schmidtea mediterranea*, a free-living flatworm (data not shown), suggesting IR-2 orthologues occur more widely among flatworms. However since this was not a full length match we could not apply this to our phylogenetic analyses. Our data combined with the previous *S. mansoni* study [26] showed that there are two types of IR present in the trematode parasites.

Sequence and structural analysis of SjIR-1 and SjIR-2 revealed that both receptors exhibit most of the features of IR family members. The extracellular domains of insulin receptors contain insulin binding sites within two characteristic loops (L1 and L2) flanked by a central cysteine-rich (CR) region (Figure 1 and Figure S1A, 1B). Identification of the binding sites of insulin to the insulin receptor is important for understanding the function of SjIRs. Recent reports indicate that the HIR has two receptor binding sites [46], [47]. The L1 sub-domain [48], [49], in which the main hydrophobic surface of insulin interacts with the hydrophobic patch in the second β –sheet of L1 [50], is one of these ligand binding sites. Our two-hybrid analysis confirmed the L1 sub-domain of the SjIRs contains the insulin binding site, as described in HIR, SmIR-1, SmIR-2 and EmIR [25], [26]. The first and second type fibronectin III repeats of the receptor contain the second ligand binding site [46], [51]. It has been demonstrated that, because of the interface between the L1 domain of one monomer and the FnIII-2 domain of the other, any closure of the L-CR unit of one monomer towards the C-terminal end of the FnIII-1 domain of the other would also drag the FnIII-2, 3 unit with it, thereby changing the relative position of the intracellular kinase domains in the IR dimer and potentially inducing signaling [46]. Homology models of the LD domains of SjIR-1 (Figure 2A) and SjIR-2 (Figure 2B) showed a similar homology modelling to the characterised LD domain of HIR (Figure 2C), implying that SjIRs may have the same predicted binding mechanisms.

Binding of insulin to the extracellular α-chains is thought to cause a change within the quaternary structure of the receptor that results in autophosphorylation of specific tyrosines in the cytoplasmic portion of the β-chains [52]. Compared with the LD domain, the TK domain is highly conserved from parasites to *Homo sapiens*. The conserved sub-domains or motifs are important for catalytic function, either directly as components of the active site or indirectly by contributing to the formation of the active site through constraints imposed on secondary structure [53]. Phylogenetic mapping of the conserved protein kinase catalytic domains can serve as a useful first step in the functional characterisation of these newly identified family members from parasites. The HIR has three sites for phosphorylation including autophosphorylation sites (YxxxYY1190), which are the key step in IR kinase activation [54]; an ATP binding site, which is utilized by many proteins which perform functions requiring an input of energy (from ATP); and a Mg^2+^ binding site, which plays a critical and conserved role in kinase function [52], [55]. Our homology models of the TK domains of SjIR-1 (Figure 2D) and SjIR-2 (Figure 2E) showed a similar structure to the characterised HIR TK domain with the same positions of conserved residues important for function, including autophosphorylation sites (YY1373 in SjIR-1; YxxxYY1413 in SjIR-2); an ATP binding site (GxGxxG); and a Mg^2+^ binding site (DFG). Despite the absence of the first Tyr of the autophosphorylation sites and the NPXY motif, the TK domain of SjIR-1 adopted a similar fold to that of HIR (Figure 2D) suggesting that the TK domain of SjIR-1 is structurally and functionally similar to that of the HIR. In SjIR-2, despite the 102aa insert, the TK domain still adopted a fold similar to that in the HIR (Figure 2E) suggesting that the 102aa insert may supplement the TK domain, providing additional functionality or interactions with another protein. Inserts also occur in SmIR-2 and EmIR, although there was no similarity between the two trematode IRs. The insert regions likely occur in loop structures, where folding allows the essential conserved regions to come together [53]. These inserts may play roles in receptor conformation adaptation or cross-talk with parallel transduction systems via their potentially phosphorylable serine and threonine sites [53]. It is noteworthy that the inserts located between sub-doamins IV and V in the TK domains of SjIR-2 (102aa), SmIR-2 (110aa) [26] and EmIR (172aa) [25], and the inserts between sub-domain I and II in the TK domains of SjIR-1 (27aa) and SmIR-1 (29aa), are absent from the IR of *Caenorhabditis elegans*. This suggests that these inserts, which lack tyrosine residues and are highly antigenic, may have functions that are unique to parasitic platyhelminth IRs and are a characteristic feature of the two types of IRs found in schistosomes. These could be considered as RTK signatures that may prove suitable as targets for the future design of specific anti-schistosome inhibitors or vaccines.

Transcription levels of the SjIRs were up-regulated in schistosomula and adult worms, further supporting their involvement in the mammalian host/parasite interaction. This might reflect differences in the demand for glucose uptake and energy requirements during the sexual (in the mammalian host) and asexual (in the snail intermediate host) reproductive phases of the life cycle of *S. japonicum*, but this needs further study. The significantly higher expression of the SjIRs in the mammalian host also substantiates the conclusions of our recent microarray analysis showing that host insulin can be exploited by *S. japonicum* for growth and development [23]. Recently, genomic analysis of signalling pathways in *S. japonicum* revealed that neither insulin growth factor nor insulin molecules were present, further supporting the notion that schistosomes exploit key signalling pathways of their hosts for growth and metabolism [21], [22]. BLAST analysis of the *S. japonicum* gene database (http://function.chgc.sh.cn/sj-proteome/index.htm) indicated there were 24 genes with high similarity to mammalian components involved in the insulin pathway suggesting it is functional in *S. japonicum*. The identification of two IRs (SjIR-1 and SjIR-2) from *S. japonicum* supports the hypothesis that these molecules are primary targets of host insulin.

The localisation of SjIR-1 in the tegument basal membrane and intestinal epithelium of adult worms, was similar to that observed with SmIR-1 [26]. Previously, tissue-specific profiling of female *S. japonicum* by laser microdissection microscopy and microarray analysis demonstrated that transcripts encoding proteins (tetraspanins, annexin and alkaline phosphatase) localised to the outer tegument of the parasite are also enriched for the gastrodermis relative to other tissues [40]. The schistosome tegument with its unusual double membrane structure plays a crucial role in the host-parasite interaction, nutrient uptake and helps protect against host immune responses [56]. SjIR-1 and SmIR-1 [26] occur in the same location in the schistosome tegument as those of the schistosome glucose transporters SGTP1 and SGTP4 [57] which are involved in the considerable uptake of host glucose, one of the major nutrients and energy substrates for schistosomes. It is likely that SjIR-1 and SmIR-1 may mimic HIR binding to host insulin on the tegument and play important roles in regulation of glucose uptake in schistosomes.

Immunolocalisation revealed that SjIR-2 is located in the parenchyma in males, which occupies most of the male tissue volume, and in the vitelline glands of female worms, which occupy 81.6% of female tissue [58] and play an important role in fecundity [59]. This suggests a putative function for SjIR-2 in providing insulin to cells inside the worm for development and/or reproduction. In contrast, SjIR-1 was located in the tegument and intestinal epithelium of adult worms, representing much smaller cellular regions compared with the voluminous vitelline tissue or parenchyma. This observation was further confirmed by real time PCR showing that SjIR-2 was more abundantly expressed in *S. japonicum* adult worm than SjIR-1. This data implied that SjIR-1 and SjIR-2 could have distinct and specialized functions. Given that two IRs have been isolated only from schistosomes to date and combined with the sequence and phylogenetic analysis and the results of the immunolocalisation studies, IR-1 may be involved in utilizing host insulin, which is a specialized function used by the parasite to exploit a host hormone, with the IR-1 homologue having been lost by other taxa during the course of evolution. The diffuse expression of SjIR-2 and SmIR-2 observed in the parenchyma of males or for SjIR-2 in the vitelline cells of females suggests their possible function in the control of growth, similar to that described for HIR [60].

Real-time PCR, using the different female tissues as templates, showed a high level of transcription of both SjIR-1 and SjIR-2 in vitelline gland tissue suggesting that both may be involved in development of vitelline for providing nutrients and shell precursors for the egg production. The high level transcription of SjIRs in vitelline tissue provides strong support for our suggestion from our earlier microarray analysis that insulin may play a role in increasing the fecundity of female parasites by activation of the MAPK sub-pathway [23]. This result is also supported by the recent finding that the proliferation, differentiation of vitelline cells and egg embryogenesis are regulated by protein tyrosine kinases [61].

The characterisation of IRs from schistosomes may have implications for understanding the host-parasite crosstalk, mediated by host hormones, activating receptors and second messenger cascades in these parasites that could lead to the design of new drugs against schistosomiasis. Recent success in cancer treatment has shown that receptor tyrosine kinases are attractive drug targets. The development of tyrosine phosphorylation inhibitors has greatly modified the approach to cancer therapy. Two possible approaches to intercepting these signaling pathways [62], which are also applicable to schistosomes are:

1. to develop biological reagents that block the binding of insulin to its receptors;
2. to inhibit the interaction of an activated intracellular tyrosine kinase with its downstream targets.

We have previously shown that the presence of insulin increases the glucose content of *S. japonicum* adult male and female worms 1.7- and 2.9-fold, respectively, *in vitro* [23]. In mammalian systems, the effect of insulin is mediated by its own receptor, but in addition, insulin can generate the formation of phosphatidylinositol 3-kinases (PI3Ks) via the IGF-I receptor [63]. HNMPA, which has no effect on the IGF-1 actions [63], was specifically devised to block insulin receptor signaling [64] and HNMPA has been found to be a specific membrane-permeable inhibitor of IR tyrosine kinase activity and non-competitive with ATP (to avoid competition with relatively high levels of intracellular ATP) [65]. HNMPA inhibits insulin-stimulated autophosphorylation of the HIR 95 kDa β-subunit that is involved in the regulation of glucose uptake in mammalian cells [64], [65]. In order to pursue our investigations on a potential role for insulin receptor signaling in schistosomes, we analysed the effect of HNMPA by measuring the glucose levels and *GTP4* transcription level of *S. japonicum* adult worms incubated with HNMPA (100 µM) for 30 min, and showed that this compound could induce a decrease of glucose levels in worms, indicating conservation between the parasite and mammalian receptors. The reason why *GTP4* transcription in adult worms increased on exposure to HNMPA can be explained by a previous investigation showing that insulin rapidly represses expression of the *GTP4* gene in 3T3-L1 mouse adipocytes, as a result of both rapid repression of transcription of the *GLUT4* gene, and an increased rate of turnover of the *GLUT4* message [66]. The results suggest that glucose uptake in the intra mammalian stages of *S. japonicum* is dependent on phosphorylation processes and could be regulated by insulin-dependent pathways, which implies that the mechanism of glucose uptake is similar to that occurring in mammals [64]. This is also supported by a recent study which showed that IGF-1 receptor tyrosine kinase inhibitors (tyrphostin AG1024 and AG538) significantly decrease glucose up-take in adult worms and schistosomulum of *S. mansoni in vitro* [67]. Tyrosine kinase and the signaling pathways in which they participate have therefore been identified as potential targets for drug design [62]. Herbimycin A, a potent inhibitor of Src kinase activities, blocked mitotic activity and egg production in female schistosomes [68] and was suggested as a novel strategy to combat schistosomiasis. As a next step, any structural divergences between the catalytic domains of the mammalian host and schistosome RTKs could be exploited for the design of inhibitor molecules which are able to target schistosomes RTKs without affecting the human host kinase.

To determine whether blocking the LDs of the SjIRs would result in changes in worm glucose uptake, we incubated adult *S. japonicum* with a mixture of the polyclonal antibodies prepared against LD1 and LD2, as these anti-sera cross-reacted with both LD recombinant proteins (data not shown). The mixture of antibodies significantly decreased glucose levels by up to 65% in adult worms *in vitro*, a result supported by a previous study whereby an anti-HIR antibody was shown capable of blocking the access of insulin to the liver receptor [69]. After 24h incubation with anti-LD anti-sera, the transcription levels of *GTP4* of treatment groups became increased significantly compared to the controls. This result is supported by a previous study showing that the level of glucose transport 4 mRNA was diminished by prolonged (24h) administration of insulin in L6 muscle cells in culture suggesting the regulation of glucose transport activity by insulin involves post-translational mechanisms and subcellular redistribution of glucose transporter proteins [70]. These results again show that *S. japonicum* IRs and the HIR have similar signaling transduction functions thereby interfering with the insulin pathway, which may affect glucose uptake in schistosomes and their growth. Blocking the LDs of the SjIRs, resulted in a significant decrease in glucose levels, and this can help explain previous work on *S. mansoni* showing that host environmental changes caused by malnutrition or by streptozotocin-induced diabetes affected the worm's ability to produce viable eggs [71], [72], which further supports our conclusion that insulin may play an important role in fecundity of female schistosmes. The specific effects of the anti-LD1 and -LD2 anti-sera remain to be determined but their overall blocking effect showed that the binding of insulin to the SjIRs is important for glucose uptake by *S. japonicum* and suggest that the SjIRs, especially the LDs, are likely targets for anti-schistosome vaccines.

Overall, our findings demonstrate that *S. japonicum* possesses insulin receptors that can specifically bind to insulin, indicating that the parasite can utilize host insulin for development and growth by sharing the same pathway as mammalian cells for the control of cell differentiation and proliferation. Further studies are necessary for precise characterisation of the signalling cascades of these receptors in schistosomes, particularly in the case of insulin-receptor signalling. Our findings have implications for the understanding of the host-parasite cross-talk and may lead to the design of vaccines or drugs that recognize parasitic molecules exclusively, with no collateral effects on the host.

## Supporting Information

Figure S1 Alignment of difference IR extracellular, intracellular regions and exon-intron structures of the SjIRs loci. The SjIR-1 and SjIR-2 extracellular (A) and intracellular regions (B) were aligned with those from *S. mansoni* (SmIR-1 and SmIR-2), *E. multilocularis* (EmIR), *D. melanogaster* (DmIR), and *Homo sapiens* (HIR) IRs. Black boxes indicate identical amino acids and grey boxes denote similarity. (A) L1, CR, L2 and FnIII1-3 sub-domains are indicated by black lines spanning the regions and the insert inside the FnIII-2 domain is shown by a dotted line. Tribasic cleavage sites, RKR857 and KK839 in SjIR-1 and SjIR-2, respectively, are underlined. Cysteine residues for the α2β2 structure in SjIR-1(C608 for α-α dimerisation; C726 and C1091 for α-β chain coupling) and in SjIR-2 (C612 for α-α dimerisation; C725 and C995 for α-β chain coupling), are shown in bold and italics. (B) A transmembrane domain indicated by a black bar. The 11 sub-domains numbered I-XI of TK domain are indicated by black lines over the regions. Crucial residues for tyrosine kinase activity are boxed in red including an ATP binding site (GxGxxG in I region), a sequence required for ATP stabilisation (VAV/IK-(16X)-E in the II and III regions), a motif for phosphotransfer (HRD LAARN in VIb region), a Mg2+ binding site (DFG in VII region), and a consensus PV/IRWMAPE sequence (in the VIII region). The insert of 102 aa between the kinase sub-domains IV and V in SjIR-2, SmIR-2, EmIR and the insert of 27aa between the sub-domains I and II in SjIR-1, SmIR-1, are shown in italics. (C) Exon-intron structures of SjIR-1 and SjIR-2 loci. Exons are numbered above the black boxes. Positions for translational start (ATG) and stop (TAA or TGA) codons are marked by arrows. The stop codon and its immediate region upstream were not amplified in SjIR-2 and are marked as “?”. Encoding regions for the different domains (A, B) are indicated by black bars.
(4.88 MB EPS)

Figure S2 Phylogenetic analysis showing relationships between SjIRs and homologues from other taxa. Phylogenetic trees of the tyrosine kinase domains (A) and ligand domains (B) for each receptor were generated as described in Materials and Methods. Values on nodes are Bayesian posterior values. Sequences other than those used in the multiple alignment (shown in Figure S1A and S1B) included: IR of helminths (SjIR-1 GQ214553; SjIR-2 GQ214554; SmIR-1 AAN39120; SmIR-2 AAV65745; *Echinoccocus multilocularis*, EmIR CAD30260; *Caenorhabditis elegans*, CeIR AAC47715), Insects (*Ixodes scapularis*, IsIR EEC19891; *Acyrthosiphon pisum*, ApIR XP_001952079; *Pediculus humanus corporis*, PhcIR EEB18223; *Tribolium castaneum*, TcIR XP_972770; *Bombyx mori*, BmIR NP_001037011; *Aedes aegypti*, AeIR Q93105; *Aedes aegypti*, AaIR EAT46545; *Drosophila melanogaster*, DmIR AAC47458), molluscs (*Biomphalaria glabrata*, BgIR AAF31166; *Lymnaea stagnalis*, LsIR Q25410; *Crassostrea gigas*, CgIR CAD59674), echinoderm (*Strongylocentrotus purpuratus*, SpIR ABC61312), sponge (*Sycon raphanus*, SrIR1 CAC14729), Hydra (*Hydra vulgaris*, HvIR Q25197), Vertebrates (*Xenopus laevis*, XiIR, CAB46565; *Gallus gallus*, GgIR, AAD26153; *Equus caballus*, EcIR XP_0014966341; *Danio rerio*, DrIR NP001136144; *Branchiostoma lanceolatum*, BLIR AAB50848), mammals (*Mus musculus*, MmIR NP_034698; *Homo sapiens*, HIR NP_000199) and of IGF-1 receptor kinase domains of mammals (*Mus musculus*, MmIGF1R Q60751; *Homo sapiens*, hIGF1R NP_000866). The catalytic domain of Homo Ros (NP_002935) and the *Mus musculus* EGFR (MmEGFR AAA17899) were used as outgroup sequences in Figure S2B. No convenient outgroup sequence was available for the tree of tyrosine kinase domains (Figure S2A), so for ease of comparison with Figure S2B, sequences from flatworms were placed at the base of the tree.
(10.20 MB TIF)

Figure S3 Yeast two-hybrid analysis. Upper panel: PCR confirming the positive colonies from medium stringency contained both the ligand domain sequences of SjIR-1 (or SjIR-2) and human insulin. Three and two positive colonies were picked up from SjIR-1 and SjIR-2 separately, the genomic DNAs were extracted and used as template (template of lanes 1, 2 from No.1 colony of SjIR-1; lane 3, 4 from No.2 colony of SjIR-1; lanes 5, 6 from No.3 colony of SjIR-1; template of lane 7, 8 from No.1 colony of SjIR-2; lane 9, 10 from No.2 colony of SjIR-2). T7 and 3′ BD primers were used to amplify human pro-insulin (558bp, lanes 2, 4, 6 from colonies of SjIR-1 and lanes 8 and 10 from colonies of SjIR-2). T7 and 3′ AD primers were used to amplify LBD of SjIR-1 (1100bp, lanes 1, 3, 5) and SjIR-2 (1270bp, lanes 7, 9). The ligand binding domains of SjIR-1, SmIR-2 and HIR were fused to the Gal4 activation domain (Gal4 AD). Human pro-insulin was fused to the Gal4 DNA binding domain (Gal4 BD). The C-terminal region of the E. multilocularis factor Elp (ElpC) was fused to Gal4 AD or Gal4 BD and used as control LBD or ligand construct, respectively. Double transformants obtained from mating of AH109 and Y187 yeast strains were assessed for colony growth after 5 days of incubation in low, medium and high stringency conditions. (++), growth in medium conditions; (-), no growth in any condition.
(5.72 MB TIF)

Table S1 Primers used for amplification of *Schistosoma japonicum* insulin receptors 1 and 2 (SjIR-1 and SjIR-2).
(0.04 MB DOC)

Table S2 Domains of *Schistosoma japonicum* insulin receptors 1 and 2 and sequence identities with other insulin receptors.
(0.03 MB DOC)
