# Supplementary figures and images for: Cloning and Characterisation of Schistosoma japonicum Insulin Receptors
Source: PLoS One. 2010 Mar 24;5(3):e9868. doi: 10.1371/journal.pone.0009868 (PMC2844434; doi:10.1371/journal.pone.0009868)

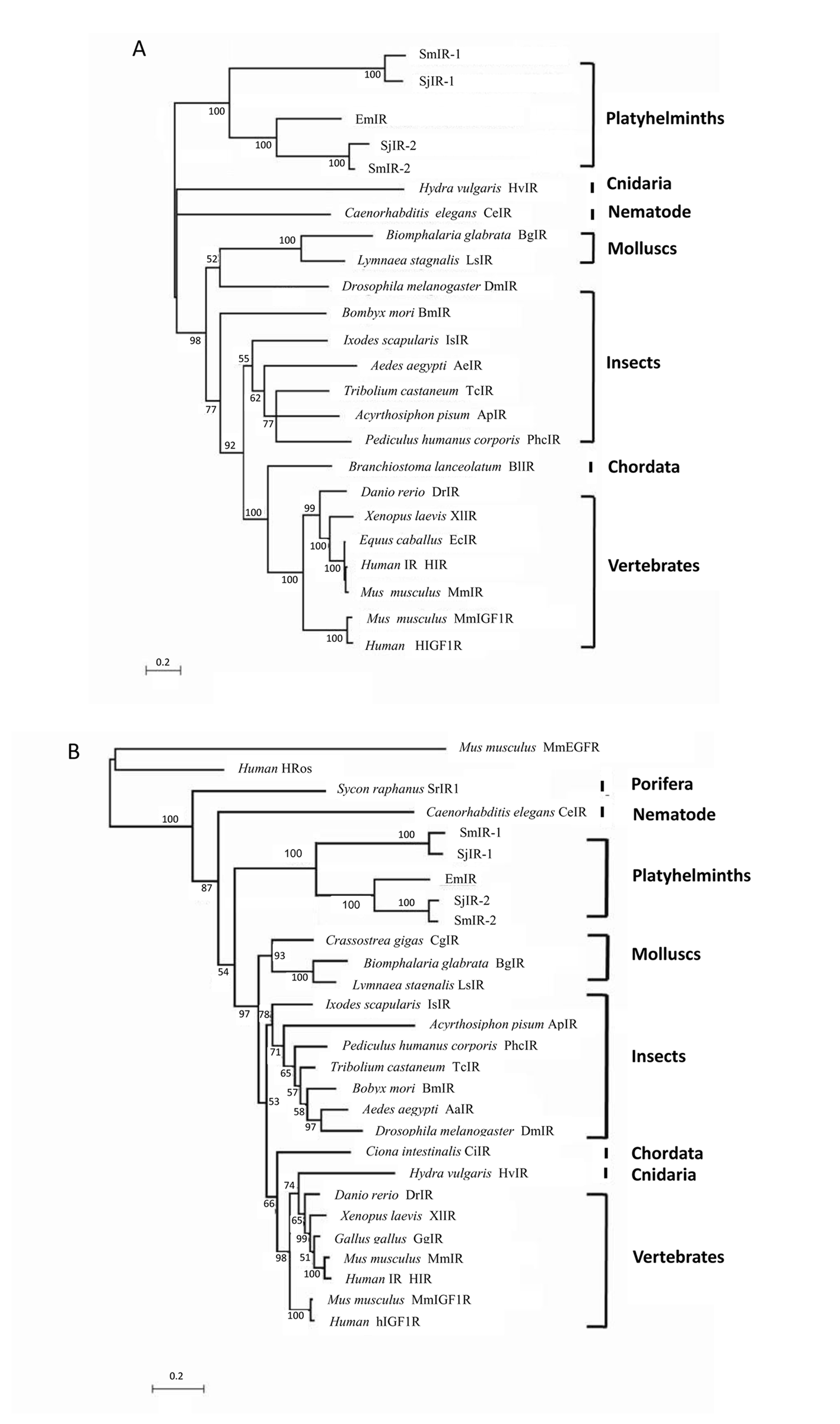

Supplement: Figure S2 — Phylogenetic analysis showing relationships between SjIRs and homologues from other taxa. Phylogenetic trees of the tyrosine kinase domains (A) and ligand domains (B) for each receptor were generated as described in Materials and Methods. Values on nodes are Bayesian posterior values. Sequences other than those used in the multiple alignment (shown in Figure S1A and S1B) included: IR of helminths (SjIR-1 GQ214553; SjIR-2 GQ214554; SmIR-1 AAN39120; SmIR-2 AAV65745; Echinoccocus multilocularis, EmIR CAD30260; Caenorhabditis elegans, CeIR AAC47715), Insects (Ixodes scapularis, IsIR EEC19891; Acyrthosiphon pisum, ApIR XP_001952079; Pediculus humanus corporis, PhcIR EEB18223; Tribolium castaneum, TcIR XP_972770; Bombyx mori, BmIR NP_001037011; Aedes aegypti, AeIR Q93105; Aedes aegypti, AaIR EAT46545; Drosophila melanogaster, DmIR AAC47458), molluscs (Biomphalaria glabrata, BgIR AAF31166; Lymnaea stagnalis, LsIR Q25410; Crassostrea gigas, CgIR CAD59674), echinoderm (Strongylocentrotus purpuratus, SpIR ABC61312), sponge (Sycon raphanus, SrIR1 CAC14729), Hydra (Hydra vulgaris, HvIR Q25197), Vertebrates (Xenopus laevis, XiIR, CAB46565; Gallus gallus, GgIR, AAD26153; Equus caballus, EcIR XP_0014966341; Danio rerio, DrIR NP001136144; Branchiostoma lanceolatum, BLIR AAB50848), mammals (Mus musculus, MmIR NP_034698; Homo sapiens, HIR NP_000199) and of IGF-1 receptor kinase domains of mammals (Mus musculus, MmIGF1R Q60751; Homo sapiens, hIGF1R NP_000866). The catalytic domain of Homo Ros (NP_002935) and the Mus musculus EGFR (MmEGFR AAA17899) were used as outgroup sequences in Figure S2B. No convenient outgroup sequence was available for the tree of tyrosine kinase domains (Figure S2A), so for ease of comparison with Figure S2B, sequences from flatworms were placed at the base of the tree. (10.20 MB TIF) [file pone.0009868.s002.tif]

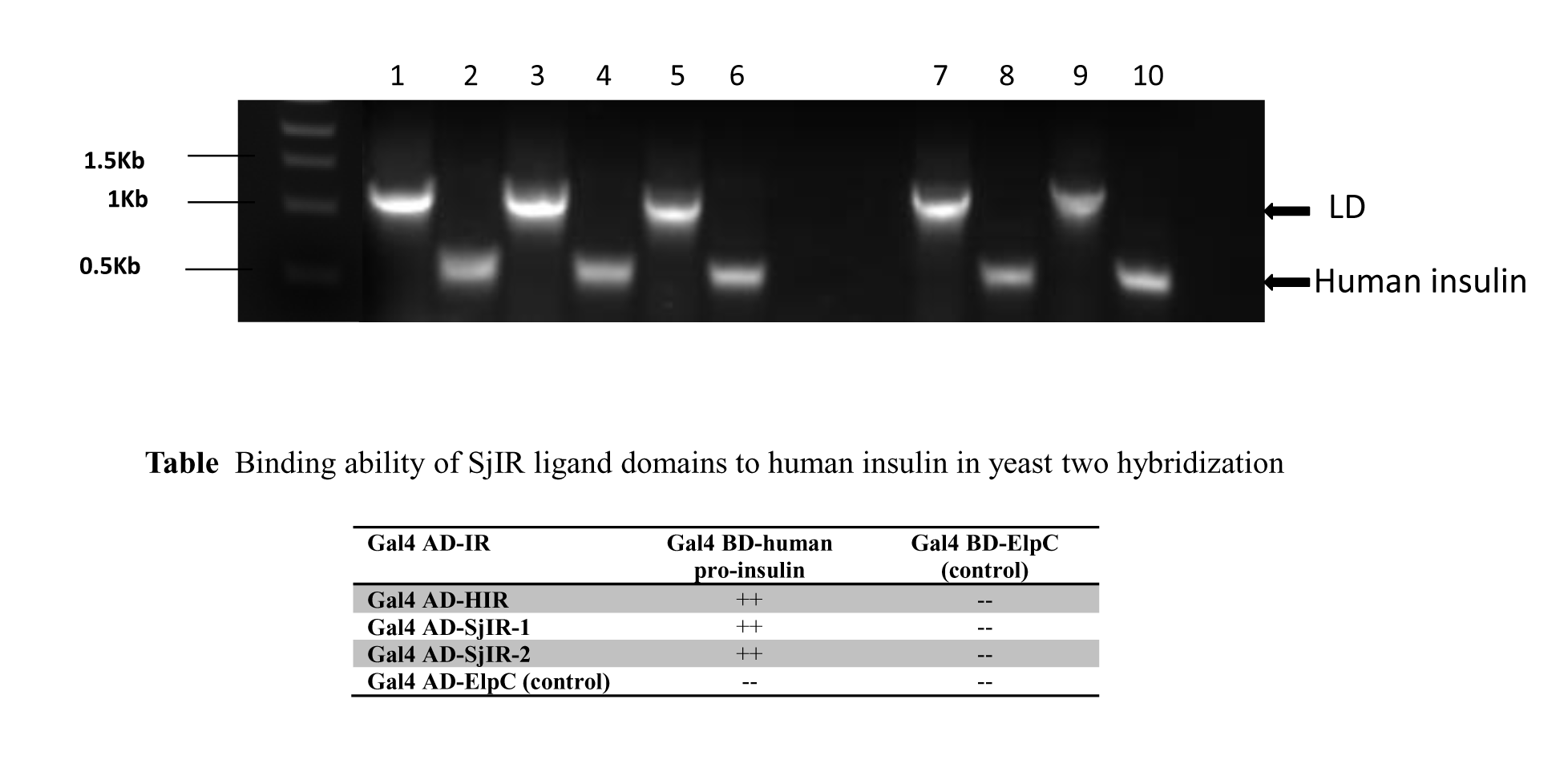

Supplement: Figure S3 — Yeast two-hybrid analysis. Upper panel: PCR confirming the positive colonies from medium stringency contained both the ligand domain sequences of SjIR-1 (or SjIR-2) and human insulin. Three and two positive colonies were picked up from SjIR-1 and SjIR-2 separately, the genomic DNAs were extracted and used as template (template of lanes 1, 2 from No.1 colony of SjIR-1; lane 3, 4 from No.2 colony of SjIR-1; lanes 5, 6 from No.3 colony of SjIR-1; template of lane 7, 8 from No.1 colony of SjIR-2; lane 9, 10 from No.2 colony of SjIR-2). T7 and 3′ BD primers were used to amplify human pro-insulin (558bp, lanes 2, 4, 6 from colonies of SjIR-1 and lanes 8 and 10 from colonies of SjIR-2). T7 and 3′ AD primers were used to amplify LBD of SjIR-1 (1100bp, lanes 1, 3, 5) and SjIR-2 (1270bp, lanes 7, 9). The ligand binding domains of SjIR-1, SmIR-2 and HIR were fused to the Gal4 activation domain (Gal4 AD). Human pro-insulin was fused to the Gal4 DNA binding domain (Gal4 BD). The C-terminal region of the E. multilocularis factor Elp (ElpC) was fused to Gal4 AD or Gal4 BD and used as control LBD or ligand construct, respectively. Double transformants obtained from mating of AH109 and Y187 yeast strains were assessed for colony growth after 5 days of incubation in low, medium and high stringency conditions. (++), growth in medium conditions; (-), no growth in any condition. (5.72 MB TIF) [file pone.0009868.s003.tif]
