# Supplementary material for: Cloning and Characterisation of Schistosoma japonicum Insulin Receptors
Source: PLoS One. 2010 Mar 24;5(3):e9868. doi: 10.1371/journal.pone.0009868 (PMC2844434; doi:10.1371/journal.pone.0009868)
Supplement: Table S1 — Primers used for amplification of Schistosoma japonicum insulin receptors 1 and 2 (SjIR-1 and SjIR-2). (0.04 MB DOC) [file pone.0009868.s004.doc]

**Table S1** Primers used for amplification of *Schistosoma japonicum* insulin receptors 1 and 2 (SjIR-1 and SjIR-2)

| Name of primers | | Sequence of primers |
| --- | --- | --- |
| SjIR-1 | IR1-1F | CGCACAAAATATTTTTTTTCGTTAAACGA |
|  | IR1-1R | CTTCACAATCACGAATACTAATAAGG |
|  | IR1-2F | AGCTTGTGGTGATATAAATGTGTATAAAG |
|  | IR1-2R | ATATATCTATAGTAAACAGTTGTAGGCA |
|  | IR1-3F | GTCGAACTCATAGACTTAATTTGTCTA |
|  | IR1-3R | GCTTCTTGTTTCATTAACCCTGAC |
|  | IR1-4F | TCTGAAACATCGTTGTCAGGGTTAATGAAACAAG |
|  | IR1-4R | CAATTTGAATAGAATTTCACTAAATGTCGG |
| SjIR-2 | IR2-1F | CTCAACATGATTCAACAGATCTCG |
|  | IR2-1R | TTTCAAATTTTTAAACGCTCTGTCCAATAAGTT |
|  | IR2-2F | TTATGCTAAATATATTGGCTCAACATGATTC |
|  | IR2-2R | GCTCGAGTCACCAATTACAATAAGCTAAATCTCCATTTGT |
|  | IR2-3F | ACAAATGGAGATTTAGCTTATTGTAATTGGCGTGA |
|  | IR2-3R | ACGACCAATTGGATATTTCCAATGTAAAACATCA |
|  | IR2-4F | TTACATTGGAAATATCCAATTGGTCGTG |
|  | IR2-4R | AAGTAGAGCGCATAGTCCTAGGAAT |
|  | IR2-5F | CTTGTCTTCCTTACTGTGGTATGTCTCATG |
|  | IR2-5R | CAGGCATGCAAGCTTTTTTTATGTGGGTCAAC |
