# Supplementary material for: Cloning and Characterisation of Schistosoma japonicum Insulin Receptors
Source: PLoS One. 2010 Mar 24;5(3):e9868. doi: 10.1371/journal.pone.0009868 (PMC2844434; doi:10.1371/journal.pone.0009868)
Supplement: Table S2 — Domains of Schistosoma japonicum insulin receptors 1 and 2 and sequence identities with other insulin receptors. (0.03 MB DOC) [file pone.0009868.s005.doc]

**Table S2** Domains of *Schistosoma japonicum* insulin receptors 1 and 2 and sequence identities with other insulin receptors

| P Positions  Domains and motifs | | SjIR-1 | Identity with SmIR-1 | Identity with HIR | SjIR-2 | Identity with SmIR-2 | Identity with EmIR | Identity with HIR |
| --- | --- | --- | --- | --- | --- | --- | --- | --- |
| Loop L1 | | N77--I199 | 73.6% | 21.4% | R36--L157 | 87% | 58.5% | 40.6% |
| Cysteine rich region (CR) | | C225--C375 | 68.2% | 21.9% | C169--C347 | 68.7% | 31.3% | 21.6% |
| Loop L2 | | G395--P506 | 84.6% | 26.7% | G367--P486 | 80% | 30.6% | 21% |
| FnIII | FnIII-1 | N514—I650 | 79.5% | 12% | E494—M694 | 69% | 18.3% | 19.2% |
|  | FnIII-2 | R651—F923  (with insert Y706—L810) | 59.1% | 18.6% | K695—H902  (with insert Y705—L829) | 69.7% | 22.5% | 17.3% |
|  | FnIII-3 | V924-I1102 | 54.7% | 24.7% | Q903--H1005 | 71.2% | 26.4% | 19.1% |
| TK domain(I-XI subdomains) | | I1176--L1476 | 74.5% | 40.8% | V1149--L1525 | 72.2% | 63.2% | 44.6% |

SjIR, *Schistosoma japonicum* insulin receptor; SmIR, *Schistosoma mansoni* insulin receptor; EmIR, *Echinococcus miltilocularis* insulin receptor; HIR, human insulin receptor; FnIII, Fibronectin 3; TK, tyrosine kinase.
